# IGF-1 Regulates Cyr61 Induced Breast Cancer Cell Proliferation and Invasion

**DOI:** 10.1371/journal.pone.0103534

**Published:** 2014-07-25

**Authors:** Suren Sarkissyan, Marianna Sarkissyan, Yanyuan Wu, Jessica Cardenas, H. Phillip Koeffler, Jaydutt V. Vadgama

**Affiliations:** 1 Division of Cancer Research and Training, Center to Eliminate Cancer Health Disparities, Department of Internal Medicine, Charles R. Drew University of Medicine and Science, Los Angeles, California, United States of America; 2 Jonsson Comprehensive Cancer Center, University of California Los Angeles, Los Angeles, California, United States of America; 3 Division of Hematology/Oncology, Department of Internal Medicine, Cedars-Sinai Medical Center, Los Angeles, California, United States of America; Sun Yat-sen University Medical School, China

## Abstract

**Background:**

Studies from our laboratory and others have shown that cysteine-rich 61 (Cyr61) may be involved in tumor proliferation and invasion. In earlier studies, we demonstrated increased insulin-like growth factor-I (IGF-1) is associated with breast tumor formation and poor clinical outcomes. In our current study we have investigated IGF-1 regulation of Cyr61 and whether targeting IGF-1 could inhibit Cyr61 induced tumor growth and proliferation.

**Methods:**

Several ATCC derived normal and breast cancer cell lines were used in this study: MDA-MB231, BT474, MCF-7, and SKBR3. We also tested cells stably transfected in our laboratory with active Akt1 (pAkt; SKBR3/AA and MCF-7/AA) and dominant negative Akt1 (SKBR3/DN and MCF-7/DN). In addition, we used MCF-7 cells transfected with full length Cyr61 (CYA). Monolayer cultures treated with IGF-1 were analyzed for Cyr61 expression by RT-PCR and immunohistochemical staining. Migration assays and MTT based proliferation assays were used to determine invasive characteristics in response to IGF-1/Cyr61 activation.

**Results:**

Cells with activated Akt have increased levels of Cyr61. Conversely, cells with inactive Akt have decreased levels of Cyr61. IGF-1 treatment increased Cyr61 expression significantly and cells with high level of Cyr61 demonstrate increased invasiveness and proliferation. Cyr61 overexpression and activation led to decrease in E-cadherin and decrease in FOXO1. Inhibition of the PI3K and MAPK pathways resulted in significant decrease in invasiveness and proliferation, most notably in the PI3K pathway inhibited cells.

**Conclusion:**

The findings of this study show that IGF-1 upregulates Cyr61 primarily through activation of the Akt-PI3K pathway. IGF-1 induced MAPK plays a partial role. Increase in Cyr61 leads to increase in breast cancer cell growth and invasion. Hence, targeting Cyr61 and associated pathways may offer an opportunity to inhibit IGF-1 mediated Cyr61 induced breast cancer growth and invasion.

## Introduction

Breast cancer mortality is due in large part to metastasis of the tumor to distant sites. Particularly, metastasis to the bone is the most common location and results in decreased patient survival [1]. Identifying biomarkers in patients at high risk of breast cancer metastasis have important therapeutic and translational implications. The CCN family of proteins have been implicated in bone metastasis in recent studies. Cyr61 is the first member of the CCN family of proteins which is named after the three proteins in the family (**C**yr61, **C**TGF, **N**OV) [2]. The more recent classification of CCN family includes: CCN1 (Cyr61), CCN2 (CTGF), CCN3 (Nov), CCN4 (WISP-1), CCN5 (WISP-2), and CCN6 (WISP-3). The CCN proteins are highly upregulated during wound healing and studies have shown that the CCN family also plays a role in tumor invasion and metastasis. Upregulation of the CCN proteins can also induce expression of a variety of angiogenic and lymphogenic factors such as the vascular endothelial growth factors [3].

The CCN family proteins share homologous binding/regulatory domains that give each protein their distinct place in the family [3]. The members of the CCN family typically have four conserved functional domains which include an insulin-like growth factor binding protein-like module (IGFBP), a von Willebrand factor type C repeat module (VWC), a thrombospondin type-1 repeat module (TSP-1), and a cysteine-knot containing module (CT). Investigations on the specific functionalities of the CCN family domains have been limited, especially on the IGFBP domain, however some reports do highlight that specific domains may play more key roles in invasiveness [4]. Importantly, it is known that the IGFBP domain on CCN1 shares homology with the traditional insulin-like growth factor 1 (IGF-1) binding proteins [3].

IGF-1 is an important modulator of cell growth, differentiation, and invasiveness [5]; thus interactions between IGF-1 and CCN1 are of significant interest. The majority of IGF-1 is found in circulation; however, in vitro studies have clearly shown that many major tissue systems including epithelial, reproductive, and cardiovascular tissues express IGF-1 and the IGF-1 receptor (IGF-1R) [6]. The bioavailability of IGF-1 is regulated by a variety of IGF binding proteins (IGFBPs). The roles of specific IGFBPs are still under investigation; however the most commonly accepted mode of action is by increasing the half-life of IGF-1 and affecting IGF-1 binding to the IGF-1R as a free ligand. Binding of IGF-1 with IGF-1R, a tyrosine kinase receptor, results in a series of intracellular signaling cascades which, in epithelial cells such as found in breast, colon, and prostate tissue, result in activation of the PI3K/Akt and MAPK pathways. The PI3K/Akt and MAPK pathways are master cell regulatory centers and subsequently promote cell proliferation, growth, and survival [7], [8].

IGF-1 has clearly been shown to be a potent mitogen in both *in vitro* and *in vivo* studies capable of breast cell tumorogenesis and breast cancer cell progression [7], [9], [10]. Evidence from clinical evaluation of systemic IGF-1 levels also reveals that IGF-1 plays a central role in breast cancer risk and outcome. Previous studies from our group and others identified that women with increased serum or plasma IGF-1 levels had increased risk for breast cancer. Patients with increased IGF-1 had poor overall survival and disease-free survival [11]–[14].Conversely, patients with positive prognosis of drug-response to therapy such as Tamoxifen concurrently exhibited reduced IGF-1 levels [11], [15]. Given the breadth of evidence implicating IGF-1 in neoplasia, several preclinical and clinical attempts have been made to develop target therapies towards IGF-1 Receptors and downstream effectors such as PI3K/Akt. Although several have reached even the Phase III clinical trial phase, results are not satisfactory and conclusive targets and therapies remain to be identified [16].

Given the known associations between Cyr61 and invasion, IGF-1 and breast cancer, and the potential interactions through the IGFBP domain on Cyr61, we investigated the role of IGF-1 on Cyr61 induced cell growth and invasion. Our earlier studies demonstrated that IGF-1 increases the risk for breast cancer and contributes to poor outcome in cancer patients [11] and pAkt is associated with poor clinical outcome in breast cancer patients [17]. Hence, we wanted to investigate if the Cyr61 levels played a role in pAkt overexpressing breast cancer cells, and to elucidate mechanisms associated with the IGF-1 axis and Cyr61 induced cell invasion. We further examined if the MAPK pathway, another key IGF-1 mediated signaling cascade, played a role in IGF-1 mediated Cyr61 expression and subsequent proliferation and invasion.

## Methods

### Cell Culture

The cell lines MCF-7, MDA-MB231, NIH-3T3 and SKBR3 cells were obtained from American Type Tissue Culture (ATCC). Cells were maintained in a T25 tissue culture flask in DMEM 50/50 1× media containing 10% Fetal Bovine Serum, supplemented with L-glutamine and Penicillin/Streptomycin at 37°C temperature [FBS (Invitrogen), L-glutamine (Invitrogen), 10 units/mL Penicillin G and 10 mg/mL streptomycin (Invitrogen)]. Additionally, altered Akt expressing subclones were utilized. SKBR3 and MCF-7 cells were stably transfected in the Vadgama Laboratory [18] with either constitutively active Akt (SKBR3/AA and MCF-7/AA) or inactive dominant negative Akt (SKBR3/DN or MCF-7/DN). The AA and DN subclones of SKBR3 and MCF-7 cells were maintained in the same growth media as the Wildtype cells, with an addition of 0.5 g/ml geneticin (GIBCO) to the culture media. MCF-7 cells stably transfected with full-length Cyr61 (MCF-7 CYA). The MCF-7 CYA clone was obtained as part of collaboration with the Koeffler Lab (Cedars/UCLA). Detailed methods on the Cyr61 transfectants have been described previously in publications from the Koeffler Lab [4], [19]. The MCF-7 cell line transfected with only the Vector (MCF-7 CMV) showed identical properties as the wild type MCF-7 cells.

### RNA isolation and Reverse Transcriptase Real Time PCR

When cells reached a confluence of 90%, the cells were collected and RNA was extracted using TRIzol reagent (Invitrogen) according to manufacturer's instructions. The cDNA was synthesized by reverse transcription (RT) with ThermoScript RT-PCR system (Invitrogen) according to the manufacturer's instructions. Real time-PCR analysis was performed with iCycle iQ real-time PCR detection system (Bio-Rad Lab, Hercules, CA) using SYBR Green Master Mix (#204143, QIAGEN). The primers used were as follows: Cyr61,5′-ACTTCATGGTCCCAGTGCTC-3′(forward) and 5′-AATCCGGGTTTCTTTCACA-3′ (reverse);18 s, 5′-GATCCATTGGAGGGCAAGTC-3′(forward) and 5′-TCCCAAGATCCAACTACGAG-3′ (reverse); E-cadherin, 5′- (ATGGGGGCTTCATTCACAT-3′)(forward) and 5′-(TTTGAGGCCAAGCAGCAG)-3′(reverse); FOXO1, 5′- (TTTGGACTGCTTCTCTCAGTTCCTGC)-3′(forward) and 5′-(TTTGACAATGTGTTGCCCAACCAAAG)-3′(reverse).

### Growth Factor and Inhibitor Induction Treatments

To determine whether IGF-1 up regulates Cyr61, MCF-7 cells were induced with IGF-1 (100 ng/ml) for 20 minutes, 4 hours, and 24 hours. Cyr61 mRNA levels were measured at different time intervals. Experimental cells were plated in 60 mm culture. Once cells reached 70% confluence they were switched to serum free media with 0.1% BSA for overnight starvation. Upon exposure to IGF-1 for the designated time the cells were spun down for RNA extraction. LY294002 (50 µM) and PD98059 (30 µM), which are PI3K and MAPK inhibitors, respectively, were used in order to determine their specific roles in response to IGF-1 mediated Cyr61 upregulation. All conditions that had a combination of IGF-1 and a kinase inhibitor were pretreated with the specific inhibitor 1 hour before the addition of IGF-1. The IGF-1 induced changes in Cyr61, E-Cadherin, and FOXO1 levels were assessed using RT-PCR and IF analysis.

### Migration Assay

Transwell plates (24-well), with cell culture inserts of 6.5 mm in diameter and 8.0-µm pore size, were used during the Migration Assay. The mouse fibroblast line, NIH3T3, was seeded on the bottom of the wells of the Transwell apparatus (25,000 cells/well). The cells were allowed to grow for two days in the normal culture media, and placed in serum-free medium (DMEM + 0.1 BSA) one day prior to the insertion of the Transwell inserts. Prior to plating onto the Transwells, breast tumor cells were disassociated into single cells and suspended in serum-free medium. Subsequently, the cells were plated on the top wells of the Transwell inserts at a density of 25,000/well (or ml) (200 µL/well). Treatments including 100 ng/mL of IGF-1, 50 µM LY294002, 30 µM PD98059 and combination of treatments, were added and the cells were allowed to migrate for 40 hours. The migratory cells on the bottom of the membrane were fixed with 5% Glutaraldehyde (Sigma) for 10 minutes at room temperature, and were then stained with 5% Toludine Blue (Sigma) and 2% NaCO_3_ (Fisher Scientific) for 15 minutes at room temperature. Excess staining was removed with water, and the chambers were air-dried. Pictures were taken under the microscope and cells numbers were quantified counting the number of migrated cells compared to the total number of cells.

### Immunofluorescence

Cells were plated for immunofluorescence (IF) onto 24 well plate circular glass inserts and fixed upon reaching 70% confluence. Fixation was performed by incubating cells with 4% Paraformaldehyde for 10 minutes followed by a two minute fixation with ice cold pure methanol. The cells were blocked with 2% horse serum diluted in 1× PBS for 1 hour, then incubated with the primary CYR61 antibody (Santa Cruz Biotechnology, Rabbit pAB 1∶400 dilution) for 1 hour. The cells were washed 3 times with 1× PBS for 5 minutes each. The cells were then incubated with secondary FITC fluorescence antibody (Santa Cruz Biotechnology, 1∶500) for one hour. Subsequently, the cells were washed 3 times with 1× PBS for 5 minutes each time, and mounted with VECTASHIELD Mounting Media with DAPI (Vector Laboratories). The cells were then imaged; FITC representing the Cyr61 protein is green, and DAPI representing the nuclear counterstain is blue.

### MTT Proliferation Assay

In order to assess growth responses of the different cells to the different treatments, MTT proliferation assays were performed. A total of 5,000 cells were plated per well in 96 well plates and allowed to attach. Cells were serum starved overnight before the addition of treatments: 100 ng/mL of IGF-1, 50 µM LY294002, 30 µM PD98059 and combination of the three treatments for 24 hrs and 48 hrs. After the treatments for the appropriate durations of time, 50 uL of MTT reagent (3-(4, 5-dimethylthiazolyl-2)-2, 5-diphenyltetrazolium bromide) was added to each well and incubated for 4 hours at 37°C. The MTT reagent was removed and DMSO was added to each well. Results were immediately read on a microplate reader (Promega GloMax Microplate Reader) at the 560 nm absorbance wavelength. All assays were performed in n = 6, and the data are presented as the mean ± SD.

### Statistical Analysis

Statistical analysis was performed using SPSS Software (version 17. IBM, Chicago, USA). Comparisons were made with the Student's *t* test and the two-tailed P-value statistic was considered. Comparisons were statistically significant if P<0.05. The comparisons from which each P-value was obtained are annotated in the figure legends.

## Results

### Cyr61 expression in different breast cancer cell types

**Figure 1:**
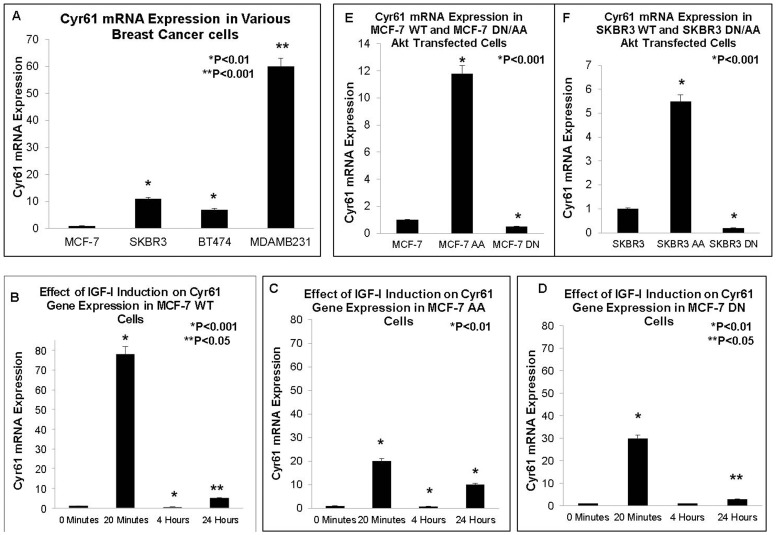
Cyr61 Levels in Various Breast Cancer Cell Lines – Baseline and in Response to IGF-1. (**A**) Baseline mRNA expression of Cyr61 in Wildtype MCF-7, SKBR3, BT474, and MDAMB231 as determined by RT-Q-PCR. (**B–D**) Cyr61 expression after IGF-1 treatment at baseline, 20 minutes, 4 hours, and 24 hours in (**B**) MCF-7 WT, (**C**) MCF-7 DN, and (**D**) MCF-7 AA cells. Cyr61 is increased by treatment of IGF-1, especially at 20 minutes. (**E**) Expression of Cyr61 mRNA in MCF-7 WT cells compared to MCF-7 AA (transfectant with constitutively active Akt), and MCF-7 DN (transfectant with dominant negative constitutively inactive Akt). (**F**) The results obtained in the MCF-7 cell line were confirmed by a second breast cancer cell line, SKBR3. All mRNA levels were adjusted for the housekeeping gene, 18S. P-values were calculated relative to MCF-7 WT in (A,E), to 0 Minutes in (B–D), and SKBR3 WT in (F). P<0.05 is statistically significant.

Baseline/constitutive levels of Cyr61 were assessed in four breast cancer cell lines each representing a specific breast cancer subtype (**Figure 1A**). These cell lines were obtained from American Type Culture Collection (ATCC). MCF-7 is an ER/PR+ and HER2- breast cancer cell line which forms localized tumors with very low invasiveness. As can be expected, MCF-7 has very low Cyr61 mRNA levels (**Figure 1A**). SKBR3 (HER2+) and BT474 (ER+/PR+/HER2+) are both HER2+ breast cancer cell lines which have some invasive properties and both express higher levels of Cyr61 than MCF-7. MDAMB231 is a triple negative receptor breast cancer cell line which is highly invasive, and has the highest levels of Cyr61 mRNA (**Figure 1A**). The P-value is <0.01 for all comparisons relative to MCF-7 WT. This study chose to focus on MCF-7 breast cancer cells since it was the least invasive line with constitutively low levels of Cyr61, and which expresses significant levels of IGF-1 receptors.

### IGF-1 regulates Cyr61 expression

The effect of IGF-1 treatment was investigated in relation to Cyr61 expression at different time-points (**Figure 1B–D**). Cells were induced with 100 ng/ml of IGF-1 for 0 minutes, 20 minutes, 4 hours, and 24 hours. In MCF-7 WT Cells (**Figure 1B**), IGF-1 induction resulted in a significant increase of Cyr61 expression, particularly at the 20 minute time point (P<0.001). We subsequently investigated whether the Cyr61 upregulation due to IGF-1 was mediated through the PI3K/Akt pathway which is one of the central pathways in IGF-1 induced cancer growth. MCF-7 clones transfected with either AA (constitutively active Akt) or DN (constitutively dominant-negative) Akt were induced with 100 ng/ml of IGF-1 (**Figure 1C and 1D**, respectively). Inactivation of the Akt pathway (such as in the MCF-7 DN cells) resulted in a significant decrease by 45% in Cyr61 levels in response to IGF-1 compared to the MCF-7 WT cells (**Figure 1D**). In the MCF-7 AA clone with constitutively high Akt activation, IGF-1 mediated increase of Cyr61 was not as significant as the MCF-7 WT, especially at 20 minutes (P<0.01 vs. P<0.001, respectively). This data suggests these cells may already have high Cyr61 and not respond as sensitively to IGF-1. These data demonstrate that IGF-1 induction results in Cyr61 expression. In addition, Akt activation is a significant contributor to Cyr61 expression; however, there also may be other pathways activated by IGF-1, such as MAPK that may also play a role. It should be noted that Cyr61 levels stabilized after 4 hours, which may suggest there may be a feedback loop that lowers the levels of Cyr61.

### Cyr61 expression is increased in breast cancer cells with activated Akt

The baseline levels of Cyr61 expression in MCF-7 WT, MCF-7 AA and MCF-7 DN were assessed to confirm that Akt activation or loss alone can regulate Cyr61 expression (**Figure 1E**). As shown in **Figure 1E**, the baseline levels of Cyr61 were significantly higher in the MCF-7 AA cells compared to the MCF-7 WT cells confirming that Akt activation alone results in Cyr61 expression (P<0.001). Furthermore, Cyr61 levels were the lowest in the MCF-7 DN cells. These trends were successfully confirmed in a second cell line, SKBR3 (**Figure 1F**), with P<0.001 for comparisons of SKBR3 AA and SKBR3 DN with the SKBR3 WT. In sum, these data suggest that Akt activation (phosphorylation) is strongly associated with Cyr61 expression. The mechanisms whereby Akt activation results in Cyr61 expression are not clearly known, however, downstream transcription factors regulated by Akt may play a role as shown later in the data.

### Cyr61 overexpression results in increased cell invasion

**Figure 2:**
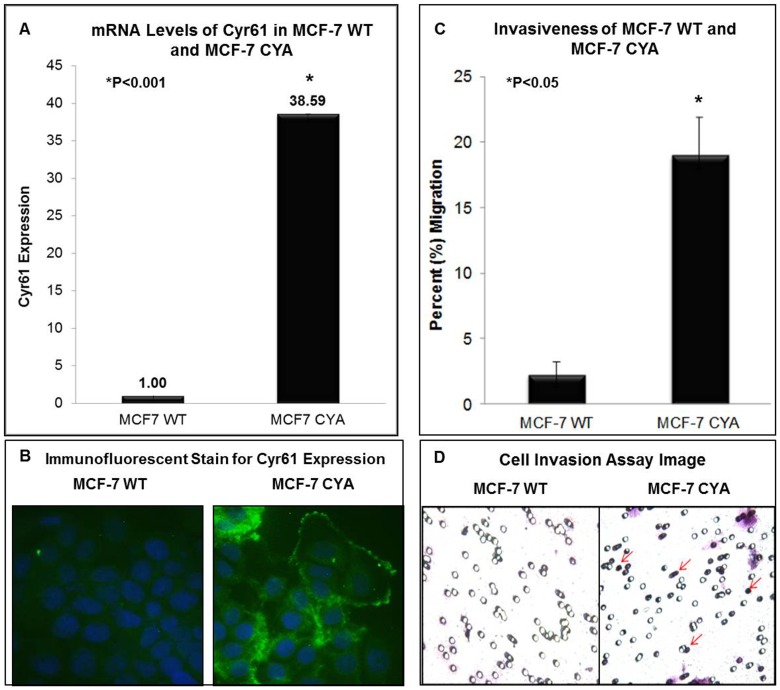
Confirmation of Cyr61 levels and Invasiveness in MCF-7 WT and MCF-7 CYA. (**A**) The mRNA expression of Cyr61 was assessed by RT-Q-PCR and (**B**) by immunofluorescent staining (FITC - green – Cyr61; DAPI – blue – nuclei). (**C**) Quantification of invasiveness from invasion chamber. (**D**) Red arrows demonstrate positive examples from invasion chamber. CYA cells are 9 times more invasive than the MCF-7 WT cells. All mRNA levels were adjusted for the housekeeping gene, 18S. P-values were calculated relative to MCF-7 and P<0.05 is statistically significant.

In order to investigate the effects of IGF-1 and its mediated pathways on Cyr61 expression, proliferation, and invasion in breast cancer, a cell model system was utilized which includes MCF-7 WT and a MCF-7 Cyr61 transfectant clone. The Koeffler lab provided the MCF-7 clone which has overexpressing full length Cyr61 (MCF-7 CYA, [19]). The MCF-7 CYA was utilized to confirm that Cyr61 overexpression alone can contribute to breast cancer cell proliferation and invasion in a non-invasive parental cell line (MCF-7). The effects of IGF-1 stimulation, and the signaling transduction pathways activated in these cells have not been investigated to date. The data in **Figure 2A** confirms that MCF-7 cells transfected with Cyr61 showed significantly increased levels of Cyr61 mRNA (P<0.001). This increase in mRNA was correlated with increase in Cyr61 protein expression, as confirmed from immunofluorescent analysis (**Figure 2B**). Consequently, Cyr61 overexpressing cells showed increased cell invasion (**Figure 2C–D**), P<0.05.

### IGF-1 induces cell proliferation and invasion via Cyr61 expression – Role of the PI3K/Akt Pathway

**Figure 3:**
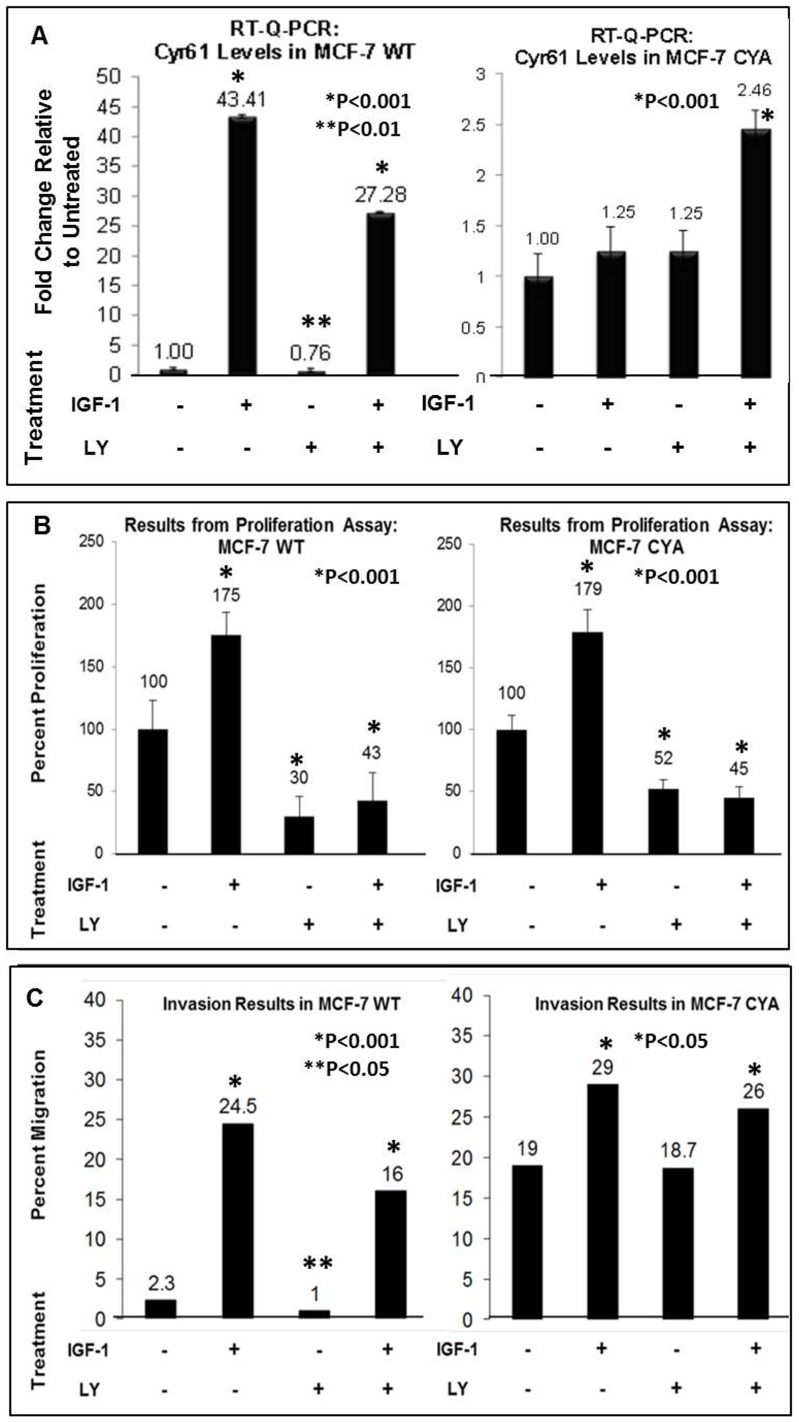
Effects of IGF-1 and LY294002 Induction on Proliferation, Invasion, and Cyr61 Expression in MCF-7 WT and CYA. (**A**) The mRNA expression of Cyr61 was assessed by RT-Q-PCR. All mRNA levels were adjusted for the housekeeping gene, 18S. (**B**) Results from proliferation assay after 48 hour IGF-1 (100 ng/mL) and LY294002 (50 µM) treatment in MCF-7 WT and MCF-7 CYA. Each condition was plated into 6 identical wells in a 96 well plate. All treatments were standardized relative to the untreated condition for each cell line. (**C**) Results from Invasion assay after 40 hour IGF-1 (100 ng/mL) and LY294002 (50 µM) treatment in MCF-7 WT and MCF-7 CYA. P-values were assessed relative to the untreated condition in each assay, with P<0.05 considered statistically significant.

Next, we examined the effects of IGF-1 treatment on breast cancer cell proliferation, invasion, and Cyr61 expression in relation to the PI3K/Akt pathway (**Figure 3A–C**). In MCF-7 WT cells, treatment of IGF-1 resulted in significant Cyr61 expression increase (P<0.001), however, blocking the PI3K/Akt pathway with LY294002 reduced Cyr61 expression significantly (P<0.01) consistent with the previous data (**Figure 3A**). Treatment of IGF-1 and LY294002 together resulted in a reduction of IGF-1 induced Cyr61 expression in MCF-7 WT (**Figure 3A**). The constitutive levels of Cyr61 mRNA in MCF-7 CYA were high. Since MCF-7 CYA is transfected with Cyr61, this is to be expected.

Both MCF-7 WT and MCF-7 CYA showed increase in cell proliferation (**Figure 3B**) upon IGF-1 treatment (P<0.001 compared with untreated in both cell lines). Treatment with LY294002 resulted in a significant decrease in proliferation in all cell types (70% in MCF-7 WT, 48% in MCF-7 CYA), confirming that PI3K/Akt plays a role. Treatment with IGF-1 in the presence of LY294002 was not sufficient to stimulate cell proliferation indicating IGF-1 mediated proliferation is significantly regulated through PI3K/Akt (P<0.001).

Migration in response to IGF-1 treatment and LY294002 inhibition was also investigated (**Figure 3C**). IGF-1 treatment induced significant increase (>10 fold) in MCF-7 WT cell migration (P<0.001). MCF-7CYA which express high levels of constitutive Cyr61 did not demonstrate as much of an additional increase in cell migration upon IGF-1 treatment (P<0.05). This implies that cells with constitutively increased Cyr61 levels are already invasive; however, they are not completely independent of further influence from IGF-1-mediated invasion.

### IGF-1 induces cell proliferation and invasion via Cyr61 expression – Role of the MAPK Pathway

**Figure 4:**
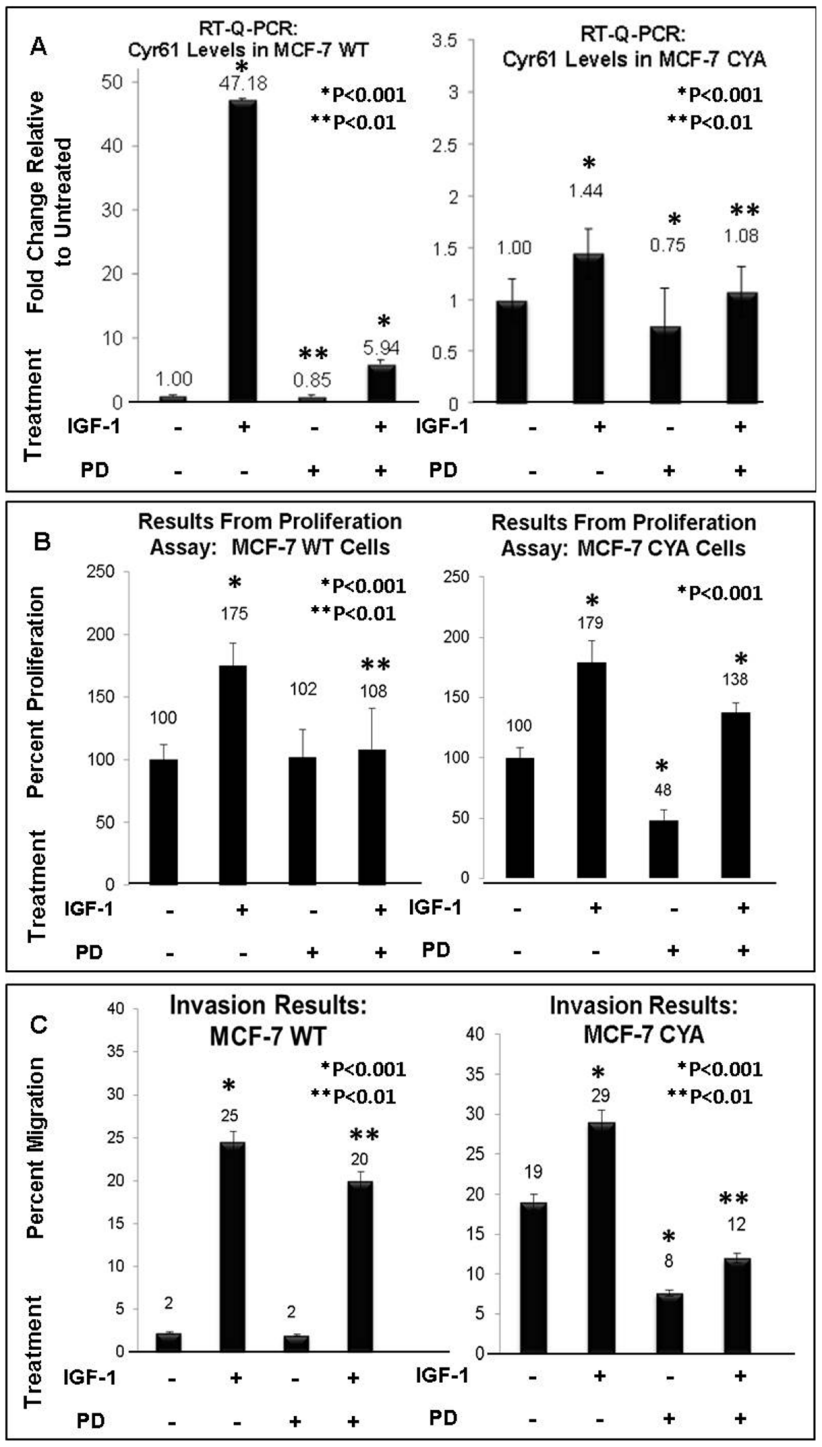
Effects of IGF-1 and PD98059 Induction on Proliferation, Invasion, and Cyr61 Expression in MCF-7 WT and MCF-7 CYA. (**A**) The mRNA expression of Cyr61 was assessed by RT-Q-PCR. All mRNA levels were adjusted for the housekeeping gene, 18S. (**B**) Proliferation assay summary data after 48 hour IGF-1 (100 ng/mL) and PD98059 (30 µM) treatment in MCF-7 WT and MCF-7 CYA. Each condition was plated into 6 identical wells in a 96 well plate. All treatments were standardized relative to the untreated condition for each cell line. (**C**) Results from Invasion assay after 40 hour IGF-1 (100 ng/mL) and PD98059 (30 µM) treatment in MCF-7 WT and MCF-7 CYA. P-values were assessed relative to the untreated condition in each assay, with P<0.05 considered statistically significant.

Cyr61 levels in MCF-7 WT cells which were concurrently treated with IGF-1 and LY294002, did not demonstrate a complete loss of Cyr61 expression when the PI3K/Akt pathway was blocked. In addition, inhibition with LY294002 did not completely inhibit IGF-1 induced cell proliferation. This data suggested that perhaps an additional pathway may be involved in IGF-1 mediated Cyr61 expression and its effect on cell proliferation and invasion. We examined the contribution of the MAPK pathway since it is one of the central pathways activated by IGF-1 (**Figure 4A–C**). **Figure 4A** shows that inhibition of the MAPK pathway with the MAPK inhibitor, PD98059, also results in a significant decrease in IGF-1 mediated Cyr61 expression (P<0.001). Again the effect was not seen as significantly (P<0.01) in the constitutively high Cyr61 expressing cells, MCF-7 CYA. In the proliferation response, interestingly, MAPK inhibition alone did not result in a significant decrease in proliferation in MCF-7 WT (**Figure 4B**). However, PD98059 did prevent IGF-1 mediated proliferation (P<0.01) in both cell lines.

In MCF-7 CYA cells (**Figure 4B**), PD98059 treatment alone did result in a significant decrease in proliferation similar to that observed in the LY294002 blocked MCF-7 CYA cells from **Figure 3B**. These data suggest that cells which overexpress Cyr61 (MCF-7 CYA) are dependent on both the PI3K/Akt pathway and MAPK pathway for proliferation. However, blocking MAPK in the presence of IGF-1 stimulation was not sufficient to attenuate proliferation to the extent as observed by blocking with LY294002 (comparing **Figure 3B** right panel vs. **Figure 4B** right panel) – P<0.001. These data suggest that inhibition of the MAPK pathway attenuates, but does not significantly diminish IGF-1 mediated proliferation as inhibition of PI3K/Akt.

When assessing migration in cells treated with PD98059 alone, there was no change in the migration of cells in the MCF-7 WT (**Figure 4C**). However, in Cyr61 overexpressing cells, MCF-7 CYA, inhibition of the MAPK pathway by PD98059 resulted in a significant decrease in cell migration (P<0.001).

### IGF-1 induction increases Cyr61 and decreases E-cadherin and FOXO1 expression

**Figure 5:**
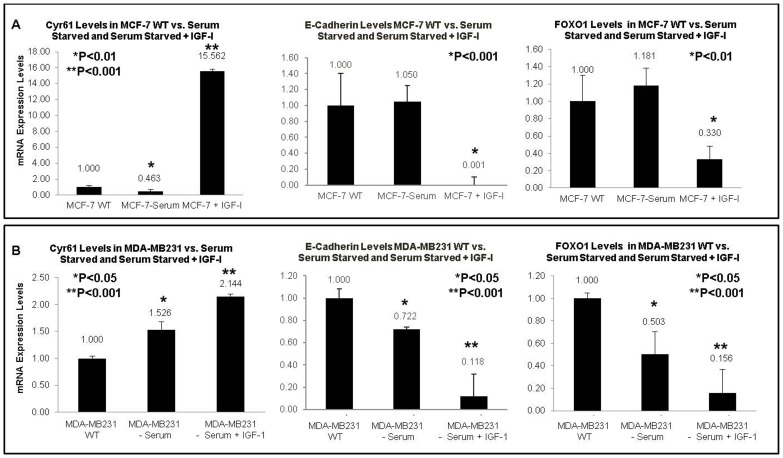
Inverse Relationship between IGF-1 induced Cyr61 upregulation and decreased E-cadherin and FOXO1 expression is confirmed in two cell lines. The mRNA expression of Cyr61, E-cadherin, and FOXO1 were assessed by RT-Q-PCR. All mRNA levels were adjusted for the housekeeping gene, 18S. (**A**) Expression of Cyr61, E-cadherin, and FOXO1 in response to IGF-1 induction (100 ng/mL) following serum starvation in MCF-7 WT cells; and (**B**) MDAMB231 cells. P-values were assessed relative to the untreated condition within each cell line, and P<0.05 considered statistically significant.

Increase in Cyr61 expression upon IGF-1 treatment resulted in increased cell invasion properties. Hence, we examined E-cadherin expression which is a key protein in cell adhesion. E-cadherin levels were assessed in MCF-7 WT (low Cyr61, non-invasive) and MDAMB231 (high Cyr61, highly invasive) cell lines in response to IGF-1 treatment. Both cell lines had a significant decrease (P<0.001) in E-cadherin in response to IGF-1 treatment (**Figure 5A–B**). Cyr61 was fairly high in these cells treated with IGF-1 (**Figure 5A–B** left panels); fifteen fold in MCF-7 and two fold in MDAMB231 (which is expected since this cell line already has very high constitutive Cyr61 levels) – P<0.001. These data indicate that upon Cyr61 increase, which is a biomarker for invasion, there is a concurrent decrease in a key cell adhesion protein, E-cadherin.

Recent studies from our laboratory [18], [20] have shown that loss or down regulation of FOXO1 is associated with aggressive cancer cell phenotype and poor prognosis in cancer patients. Furthermore, FOXO1 is a downstream target of the PI3K/Akt pathway, which we have identified affects Cyr61 expression and proliferation. Hence, we examined if IGF-1 treatment caused deregulation of FOXO1 in association with increase in Cyr16. **Figure 5A–B** (right panel) demonstrates that increased Cyr61 levels in response to IGF-1 resulted in decrease in FOXO1. Both MCF-7 and MDAMB231 showed a significant decrease in FOXO1 levels - over 60% in MCF-7 (P<0.01) and over 80% in MDAMB231 (P<0.001). These data indicate two potential mechanisms whereby IGF-1 induced proliferation and invasions may occur – deregulation of FOXO1 and loss of E-cadherin. Additional research is needed to investigate the mechanisms linking FOXO1 and E-cadherin with Cyr61.

## Discussion

Identification of biomarkers which play a clear functional role in breast cancer etiology and invasion will be key for improving breast cancer outcomes. Notably, these biomarkers have the potential, with additional validation, to one day possibly inform therapeutic options for breast cancer patients. Breast cancer metastasis to the bone is a common metastatic event associated with reduced survival. Bisphosphonates have been shown to prevent bone metastasis and reduce tumor burden in several preclinical [21] and clinical models [22]. Extracellular matrix proteins related to invasion in tumors, such as the CCN family which are responsive to well-established growth factor signaling implicated in cancer, are strong candidates for molecular examination. The CCN family of proteins play a role in so many key molecular processes [2]. They are defined by their various homologous domains – including the IGF Binding Protein domain which is of particular interest given that previous studies in our lab and others have identified that IGF-1 serum levels are associated with breast cancer tumorogenesis and progression [11], [12]. In addition, studies have also shown that patients with elevated expression of CCN1 in their breast cancer have a worse prognosis [19], [23]. Hence, these pathways and interplay of the IGF-1 axis with the CCN1 expression is of particular interest and has important translational potential.

In the present study, the effect of IGF-1 induction on Cyr61 activity and subsequent cell proliferation and invasion was examined. The study identified that IGF-1 induced an increase in Cyr61 levels in MCF-7 WT cells which resulted in both increased proliferation and invasion. This was confirmed by the transfection of Cyr61 full-length protein into MCF-7 (MCF-7 CYA) which also resulted in significant increase in proliferation and invasion as compared with WT. Previous studies by Xie et al [19], [23], and Tsai et al [24] confirm that increased Cyr61 in breast cancer cells result in aggressive properties. Together, our studies indicate that Cyr61 is inducible both by estrogen as well as IGF-1, rendering this molecule highly sensitive to growth factor induction. Physiologically, patients with high IGF-1 circulatory levels have been shown to have reduced survival from breast cancer; hence, the upregulation of Cyr61 which mediates cell migration may result in increased metastasis observed.

The mechanism whereby IGF-1 stimulates Cyr61 was also investigated in the present study. The PI3K/Akt pathway was assessed given that it is one of the central downstream pathways from IGF-1 and previous studies from our group and others identified a central role of PI3K/Akt in breast cancer outcomes [17]. Transfection of constitutively active Akt resulted in significant increase in Cyr61 expression, whereas inactive Akt transfection resulted in a decrease. This was further confirmed by inducing the Akt pathway with IGF-1 and blocking the Akt pathway with LY294002. The cell proliferative activity and invasion mirrored Cyr61 transcriptional levels. However, the IGF-1 induced transcription of Cyr61 was not completely abolished. Therefore, MAPK the other key downstream pathway from IGF-1 was assessed. Inhibition of MAPK resulted in a significant decrease in Cyr61 expression in the WT cells which was not restored by IGF-1 induction, unlike the response seen by blocking the PI3K/Akt pathway. However, similarly to the LY294002 response, blocking MAPK with PD98059 reduced proliferation and invasion. Together these data suggest that IGF-1 mediated Cyr61 expression, proliferation, and invasion are intrinsically but not exclusively mediated through MAPK and PI3K/Akt signaling with high Cyr61 expressing cells less sensitive to MAPK inhibition than PI3K/Akt inhibition in presence of IGF-1.

Furthermore, we posit it is the activation of Akt (pAkt) rather than the expression of Akt that is the driver of proliferation and invasion in our model. When the PI3K inhibitor is applied, we see loss of the growth and proliferation because phosphorylation of Akt is inhibited, rather than the actual expression. In preliminary evaluation of the cell lines, we identified that baseline levels of pAkt were higher in MCF-7 CYA cells than MCF-7 WT cells (data not shown). These data are consistent with our previous findings indicating that aggressive breast cancer phenotypes do not necessarily have increased levels of Akt, but do have increased levels of active Akt (pAkt) [17]. This finding was further confirmed in breast cancer patient clinical samples; patient tissues with high pAkt levels had reduced disease free survival [17].

Previous studies have highlighted the role of estrogen-induced Cyr61 expression in breast cancer [19], [25]. However, the presence of the estrogen receptor (ER) is implied to be present in these cell lines. Paradoxically, Cyr61 is most highly expressed in ER- cells lines such as MDA-MB231 as reported previously [19], [26] and confirmed in this present study. The finding from our study implicating IGF-1 in Cyr61 expression and subsequent proliferation and invasion can shed light on this paradox as well as present several translational implications. As indicated earlier, IGF-1 levels have been associated with more aggressive breast cancers resulting in reduced disease free survival and reduced response to breast cancer treatment [11]. Furthermore, IGF-1 levels are higher among African-American women [11], [27], [28] and triple negative breast cancer is more frequent among African-American women [29]. Therefore, it is possible that increased IGF-1 induced Cyr61 activity among these cohorts through classical PI3K/Akt and MAPK signaling is a mechanism whereby these tumors progress to a more aggressive and invasive phenotype. These data were preliminarily confirmed by our study which identified the corresponding reduction of E-cadherin expression in response to IGF-1 induction and Cyr61 expression. E-cadherin is a well-established “gate-keeper” for cell-adhesion and loss has been associated with epithelial mesenchymal transition and increased invasiveness [30]–[32]. Loss of E-cadherin in response to Cyr61 has been shown in Cyr61 induction studies in gastric cancer [33], however, we are among the first to report these findings in breast cancer.

Furthermore, the high IGF-1 and Cyr61 levels may play a role in therapy and drug-resistance. There is data to support this for patients with high IGF-1 levels [11], [15]. In vitro studies by Menendez et al demonstrated Cyr61 overexpression resulted in Taxol resistance [26] which was further confirmed by Lai and colleagues [34]. These data add an additional layer of complexity related to lack of therapeutic response and progression of the tumor to metastasis in Cyr61 overexpressing cells. Previously, studies from our group identified the forkhead-family member, FOXO1, to play a role in drug-resistance and survival in breast cancer [18], [20]. Our present study presents preliminary data that FOXO1 was downregulated in response to IGF-1 induction and associated with Cyr61 expression. Hence, Cyr61 expression may be a key molecule, mediated through IGF-1, which contributes to drug resistance and the aggressive breast cancer phenotype. Additional studies are needed to confirm these findings. Early phase clinical trials have already attempted to directly target IGF signaling through anti-IGF receptor (IGF1R) antibodies with limited success [16]. Hence, targeting Cyr61 directly or through one of the downstream signals may offer alternative therapeutic options.

Direct Cyr61 targeting has been attempted recently by several groups. Lin and colleagues have just developed a mAb termed 093G9 which targets the VWC domain of the Cyr61 and reduces MDA-MB-231 cell proliferation, migration, and invasion [35]. Similarly, Jim Leu et al recently developed a mAb termed YM1B targeting the DM region of Cyr61 which has successfully attenuated cell migration/invasion through the integrin/Rac1/ERK signaling axis [36]. These are undergoing additional validation and not yet have reached clinical testing. Therapies targeting the Cyr61 binding partners such as integrins may offer additional strategies. Cyr61 has been shown to bind with integrins such as αvβ5 and α3β5, in the extracellular matrix space [37]. Integrin binding has been shown to induce actin filament rearrangement which can contribute to morphological changes- such as epithelial-mesenchymal transition [33]. Menendez and colleagues have demonstrated in several studies that targeting the αβ integrins in Cyr61 overexpressing breast cells successfully restores drug-response [26], [38]. This may be another therapeutic target to investigate further for attenuating Cyr61 mediated cell activity and drug resistance.

**Figure 6:**
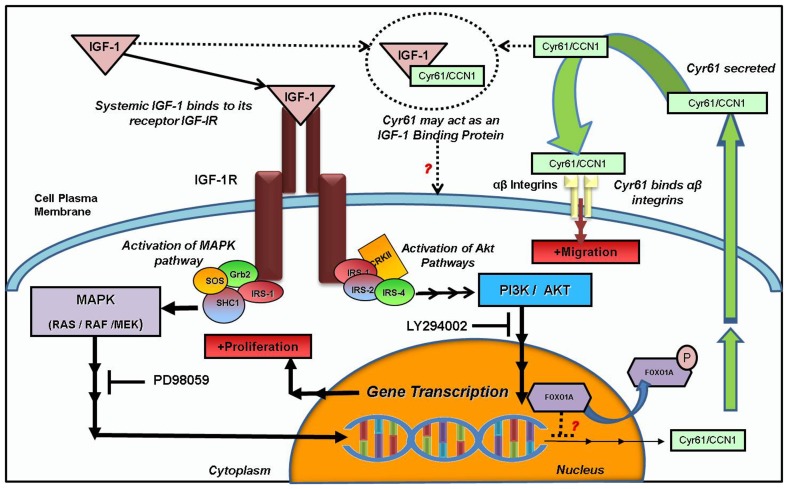
Proposed Schematic of IGF-1 mediated signaling in relation to Cyr61 expression and activity.

In summary, this study demonstrates that IGF-1, which is significantly involved in poor prognosis and outcome in women with breast cancer, upregulates Cyr61 through both the MAPK and PI3K/Akt pathways resulting in cell invasion and growth (Figure 6). Furthermore, IGF-1 mediated cell invasion and growth are accompanied by the loss of E-cadherin and FOXO1, key biomarkers regulating EMT/cell-adhesion and drug response, respectively. Thus, one of the possible mechanisms whereby high levels of IGF-1 among patients may result in poor outcome and prognosis may be due to the activation of Cyr61 and downregulation of the other protective biomarkers. Since IGF-1 is a growth factor essential for maintaining systemic homeostasis in key tissues such as the muscles and heart, targeting of IGF-1 may not be feasible for attenuating cancer risk and/or progression. Thus, targeting downstream players, such as Cyr61 may be more realistic potential therapeutic objectives for invasive breast cancer cells.
